# Porcine salivary carbonic anhydrase VI is involved in the pathogenesis of disease

**DOI:** 10.3389/fvets.2025.1675415

**Published:** 2026-01-07

**Authors:** M. Matas-Quintanilla, R. I. López-Balibrea, I. Miller, A. M. Gutiérrez

**Affiliations:** 1 BioVetMed Research Group, Department of Animal Medicine and Surgery, Veterinary School, CEIR Campus Mare Nostrum (CMN), University of Murcia, Murcia, Spain; 2 Department of Biological Sciences and Pathobiology, University of Veterinary Medicine Vienna, Vienna, Austria

**Keywords:** carbonic anhydrase VI, disease, health status, pig, saliva

## Abstract

**Introduction:**

Evidence exists of the role of salivary carbonic anhydrase VI (CA VI) in the protection of the oral mucosa and in the gastrointestinal and respiratory tracts in different species. However, little is known of the behaviour of CA VI in porcine disease. We aimed to study the behaviour of salivary CA VI in porcine stress and disease for the first time, and to study the concentrations of the CA VI in different homeostasis disturbances under field disease conditions.

**Material and methods:**

We used saliva samples from 30 healthy pigs and 30 pigs with different disorders in a validation trial using protein separation and specific CA VI detection. Afterwards, the salivary CA VI concentrations of 255 clinically healthy pigs and 371 pigs with different signs of disease were quantified using a new in-house immunoassay in a field trial in which salivary alpha amylase and C-reactive protein (CRP) levels were also evaluated.

**Results:**

The overall specific CA VI signal appeared increased in disease conditions in comparison to the healthy state, whilst stress showed no statistical modification. The immunoassay developed for CA VI quantification showed good analytical performance and revealed increased concentrations in all the diseases studied in comparison to healthy animals, with pronounced intensities in gastrointestinal and respiratory diseases, followed by animals with inflammatory conditions. CA VI showed a moderate positive association with alpha amylase and a low but positive association with CRP.

**Discussion:**

The present findings suggest that salivary CA VI is involved in the pathogenesis of disease in pigs with an increase in its concentrations; however, the specific roles attributed to CA VI in disease conditions should be further studied.

## Introduction

Carbonic anhydrases are an enzymatic group containing zinc in their active centre, which is responsible for catalysing the hydration of CO_2_ to carbonic acid, which spontaneously turns into bicarbonate, under the release of protons to the medium (1, 2). Currently, 16 different isoforms are known in mammals, and they are distributed throughout different tissues and organs (2). Specifically, CA VI is the only secreted isoenzyme of this family in mammals (3), although different studies claim to have detected the secretion of CA II in the saliva of different ruminants, as well as in primates, by the parotid gland (4, 5). The protein CA VI has a molecular weight (MW) of approximately 40 kDa in human saliva (6) and is normally glycosylated, with two putative N-glycosylation sites. CA VI was purified from swine saliva with a molecular weight of 250 kDa, and the molecule consisted of 7 subunits of 37 kDa (7), which after deglycosylation with endo-N-acetylglucosaminidase F, decreased in molecular weight to 35 kDa and/or 32 kDa (7). The degree of glycosylation, as well as phosphorylation, can influence the molecular weight and isoelectric point (pI) of proteins, potentially altering their detection (8) and could be the reason for their identification at different sizes. Accordingly, we have previously identified in several proteomics studies CA VI at different positions, approximately 36 kDa and 33 kDa in porcine salivary profiles (9–13).

CA VI is secreted by the parotid gland with a higher contribution to whole saliva after chewing stimulation (14). Its secretion pattern varies depending on circadian cycles, and its periodicity is very similar to that of salivary alpha amylase in humans (3). Most authors agree that the main function of CA VI, a result of the reaction it catalyses, is to act as a modulator of the acid–base balance in different tissues and secretions (15), such as in saliva (16), urine (17), and cornea (8). More specific functions would be, for example, participation in the function of taste (18), in the control of the oral (19) and rumen microbiota (5), homeostasis of the oral cavity and dental protection (16), or the stabilisation of sperm for correct fertilisation (15).

A protection role has been defined for salivary CA VI in the gastroesophageal mucosa (20), stomach, and duodenum (21), and in the lower respiratory tract (22).

The literature about porcine salivary CA VI protein alterations did not show uniform behaviour. Homeostasis alterations can be accompanied by increased, decreased, or unchanged salivary CA VI. For example, one study found increased CA VI in the non-glycosylated form at 31 kDa in pigs after acute stress (11), whilst no alterations have been observed under chronic stress (23). Other studies showed decreases in the glycosylated fraction of CA VI at 36 and 33 kDa in pigs suffering from rectal prolapse in comparison to healthy pigs (12), whilst a decrease at 33 kDa was accompanied by an increase at 36 kDa in animals with non-infectious growth rate retardation (13). One reason for this inconsistency of results may relate to the proteomics nature of the studies, in which the analysis showed alterations in specific CA VI forms with different degrees of glycosylation. There is only one study in which a competitive assay was developed to measure the levels of CA VI in different porcine body fluids, including saliva, in a small number of animals, specifically 16 pigs aged 4–5 years (15). More recently, a study reported increased esterase activity under disease conditions, in which CA VI is implicated (24). However, to the authors’ knowledge, no studies have evaluated the global regulation of whole salivary CA VI levels under field disease conditions in pigs. We hypothesised that if salivary CA VI is involved in the restoration of homeostasis in the pig:

(i) Disease conditions would produce variations in the global levels of salivary CA VI that could be quantified in the saliva of pigs and (ii) the degree of variations in the concentrations of salivary CA VI is not identical in all diseases and could be attributed to different roles of the protein. Hence, the aim of the study is to: (1) obtain a first approach to the overall behaviour of salivary CA VI under stress and disease in pigs and (2) quantify the concentrations of salivary CA VI from pigs under different homeostasis alterations in field conditions.

## Materials and methods

### Reporting

Material and methods are reported according to “The ARRIVE Guidelines for Reporting Animal Research.”

### Study design

#### Validation trial

A total of 60 saliva samples, from 10 healthy pigs and 10 pigs under pathological conditions, from 3 different farms in the southeast of Spain were used for the validation trial. The pathological conditions included were rectal prolapse at farm 1, tail biting at farm 2, and acute stress due to nasal restraint and blood sampling at farm 3. All saliva samples were obtained from previous studies [Sánchez et al. (25), for prolapse and tail biting animals & Gutiérrez et al. (11), for stressed animals, with their healthy counterparts]. The validation study was performed on aliquots of the fresh saliva samples stored at −80 °C within 2 months after collection.

Total protein content was measured on individual saliva samples prior to electrophoresis, as reported before (26). In brief, the absorbance of the mixture of 50 μL of 1:40 diluted saliva with 0.9% sodium chloride and 200 μL of Bradford reagent was measured at 590 nm in a microplate reader (Spectro Star Nano, BMG Labtech, Ortenberg, Germany) and extrapolated into a four-parameter calibration curve constructed with bovine albumin ranging from 5 to 100 μg/mL. SDS-PAGE was performed with 5 μg of total salivary protein in 12% polyacrylamide homemade mini-gels in a vertical electrophoresis chamber (Mini-PROTEAN Tetra Vertical Electrophoresis Cell, Bio-Rad, Hercules, CA, United States) as reported before (13).

After electrophoresis, salivary proteins were electroblotted into PVDF membranes using the Trans-Blot Turbo Transfer System (Bio-Rad, Hercules, CA, United States) for western blotting analyses. Membranes were incubated with a goat-anti-human CA VI antibody (carbonic anhydrase VI polyclonal antibody, Invitrogen, Thermo Scientific, Rockford, IL, United States) at 0.4 μg/mL, followed by a horseradish peroxidase-conjugated rabbit anti-goat IgG (Sigma Aldrich, St. Louis, MO, United States) secondary antibody at 0.16 μg/mL. The specific CA VI signal was detected by enhanced chemiluminescence (ECL 2 Western Blotting Substrate, Thermo Scientific, Rockford, IL, United States) in an imager LAS 600 (GE Healthcare, Uppsala, Sweden). The band volume signals of the different saliva samples were recorded using image analysis software (ImageQuant TL v2005, Amersham Biosciences Europe GmbH, Freiburg, Germany). For the band volume signals comparison between healthy pigs and pigs under pathological conditions (rectal prolapse, tail biting, and stress), the band volume signal intensity of the internal control sample was used for data normalisation.

#### Field trial

The minimal sample size needed was estimated based on the results of the validation trial using specific statistical tools (G*Power). The analysis suggested a sample size of 33 independent animals per group for an effect size of 0.9, a power of 95%, and an error of 0.05. An extra 20% was added to account for missing values or outliers (the minimal sample size required was 40 pigs per group).

Six groups of animals were formed: healthy pigs, animals suffering from tail biting, respiratory disorders, gastrointestinal diseases, inflammatory conditions, or pigs with mixed pathologies. A range of 15 and 40 diseased animals per farm was sampled. A minimum of 40 healthy pigs, 20 males and 20 females, per farm were sampled from a total of 6 farms. For sampling diseased pigs from 6 different farms, 12 days were spent, whilst the sampling procedure from healthy animals was performed in 6 days. A total of 255 clinically healthy pigs and 371 pigs with signs of disease were enrolled in the field trial (Supplementary Table S1).

Pigs were allocated to one of the groups based on clinical examination. Those pigs without any clinical signs of disease were included in the healthy group (*n* = 255). When pigs showed acute tail wounds or when there was evidence of swelling/abscessation around the base of the tail, pigs were allocated to the tail-biting group (*n* = 74). The group of pigs with respiratory signs was composed of animals with dyspnoea, respiratory distress, cough, and/or discharge from the nose with or without cachexia (*n* = 91). The gastrointestinal group was characterised by animals with clinical signs such as diarrhoea, vomiting, and/or rectal prolapse with or without growth retardation (*n* = 85). Pigs with inflammatory conditions were those animals with lameness, arthritis or osteoarthritis, superficial abscesses, or ear wounds (*n* = 79). The mixed group included pigs with a combination of pathological conditions, specifically animals that showed signs of more than one group amongst the above (*n* = 42).

All pigs were commercial pigs from 11 farms, belonging to the same commercial company in the southeast of Spain, and were housed in pen groups with 0.65 m^2^ per pig following the official standards (27) with white LED lighting. All pigs had ad libitum access to balanced standard commercial dry food (for details on feed composition, see Supplementary Table S2). The mechanical nipple drinker provides freely accessible well water for the pigs. Cleaning and disinfection of the porcine facilities were performed similarly in all farms before the introduction of a new group of pigs. The temperature control was performed by manual control in 6 farms and by automated control in 5 farms; no humidity control was established at any of the farms in the study, and the ventilation control was manual for 10 farms and mechanical in 1 farm. All farms followed a similar vaccination programme, which consisted of vaccination against *Mycoplasma hyopneumoniae* and *Porcine circovirus type 2* before weaning; in the nursery, pigs received the first dose against *Actinobacillus pleuropneumoniae* and at growing, they received the second dose and the two doses against Aujezsky’s disease. Clinical examination of animals was performed and annotated by the official veterinary personnel before sample collection.

Saliva samples were collected individually by allowing the pigs to chew a sponge clipped to a thin metal rod for approximately 1 min. Sponges were then placed in specific tubes (Salivette tubes, Sarstedt, Germany) and refrigerated in cooling accumulators. In the laboratory, no longer than 4 h after sampling, the tubes were centrifuged at 3000 g for 10 min (Fisherbrand GT2R Expert Centrifuge, Thermo Fisher Scientific, United States). Salivary supernatants were stored in 1.5 mL tubes at −80 °C until analysis. The concentrations of CA VI, alpha amylase, and CRP in all the individual saliva samples were quantified.

### Salivary quantifications

A sandwich enzyme-linked immunosorbent assay (ELISA) was developed for CA VI quantification using rabbit monoclonal anti-human CA VI antibodies (Human carbonic anhydrase VI antibody pair, Abcam, Netherlands). The assay used 3 μg/mL of capture and biotinylated detection antibody and a dilution of the saliva samples of 1:5. A calibration curve with seven standards ranging from 160 to 2.5 ng/mL was constructed using human recombinant protein (Abcam, Netherlands) and a four-parameter curve from which the samples’ concentrations of CA VI were finally obtained, based on their measured absorbance values. The absorbance was recorded at 405 nm in a microplate reader (Spectro Star Nano, BMG Labtech, Ortenberg, Germany) after the incubation of the detection antibody for 1 h with Streptavidin-HRP (Ultra Streptavidin-HRP, Thermo Fisher Scientific, United States) and the development of signal after 25 min incubation with ABTS substrate solution.

A basic analytical validation of the assay developed for CA VI quantifications was performed, including the calculation of the intra- and inter-assay precision, the accuracy, and the limit of detection, following standard guidelines (28).

For the quantification of alpha amylase levels, an optimisation of a commercial assay was used (salivary *α*-amylase kinetic enzyme assay kit, Salimetrics, United States) (26). To sum up, the assay consisted of the reaction of 8 μL of undiluted saliva samples with a total of 320 μL of amylase reagent. The absorbance levels at 405 nm were measured at 1 and 6 min of incubation at 37 °C, and afterwards the increase in the absorbance was calculated. The levels of alpha amylase in the saliva samples were expressed as U/L according to the manufacturer’s instructions (U/L = Δabs*655). When the values were above the upper limit of the assay, the saliva samples were reanalysed at 1:4, 1:8, or 1:16 dilutions.

The concentration of the acute phase protein CRP in the saliva samples was measured using a previously validated time-resolved immunofluorometric assay (29) in undiluted saliva samples.

### Statistical analysis of data

In the validation trial, all normalised band volume signal intensities were analysed for outlier identification using the ROUT method and removed from the raw data. Afterwards, distribution and homoscedasticity criteria were tested using the Shapiro–Wilk and Bartlett’s test, respectively. Five bands were evaluated corresponding to different molecular weights; specifically, band 1 corresponds to 216 kDa, band 2 to 66 kDa, band 3 to 35 kDa, band 4 to 32 kDa, and band 5 to 30 kDa. Moreover, analysis was also performed for the summed-up data from all CA VI bands per lane. For data not following normal distribution criteria but homoscedasticity criteria, the Kruskal–Wallis test with Dunnett’s multiple comparisons test was used (bands 1 and 2), whilst for data that followed normal distribution criteria but following not homoscedasticity criteria, the Brown–Forsythe and Welch ANOVA tests with Games–Howell’s multiple comparisons test were performed (bands 3, 4, 5, and total bands).

In the field trial, several statistical analyses were performed. Normality and homoscedasticity criteria were tested as detailed above before outliers’ identification using the ROUT method. Initially, the concentration of CA VI between healthy animals of the growing and finishing stages was compared using Mann–Whitney *t*-test; as the results showed no statistical differences, healthy animals of both stages were grouped in a unique healthy group of pigs for the remaining analyses. Then, to discriminate between healthy and diseased animals, the concentrations of CA VI, alpha amylase, and CRP were compared between both groups using the unpaired *t*-test with Welch’s correction. Afterwards, the possible association between the quantifications of the three parameters was evaluated using a Spearman’s correlation test. Finally, the behaviour of the levels of CA VI, alpha amylase, and CRP in the different groups of diseased animals in comparison to the group of healthy pigs was evaluated using a Brown–Forsythe and Welch ANOVA test and to analyse the magnitude of the effect of the variations in every disease in comparison to the healthy group, specifically the size effect, the Cohen’s d for independent groups was individually calculated.

All the statistical analyses were performed using the statistical software GraphPad Prism, version 10.4.2, except for the effect size, which was calculated using jamovi software, version 2.6.44.

For the analytical validation of the CA VI assay developed, the precision was analysed by the calculation of the intra- and inter-assay coefficient of variation in % by the formula: mean/standard deviation * 100. The accuracy was assessed by linear regression analysis of the observed and expected CA VI concentrations in serially diluted saliva samples by the calculation of the coefficient of determination (*R*^2^). The limit of detection was defined as the CA VI value of a zero sample and was evaluated by measuring 10 replicates of a blank using the formula: mean + 2 * SD according to the reference guide (28). The calculation of the coefficients of variation, the coefficient of determination, and the limit of detection was performed using Microsoft Excel software.

## Results

### Validation trial

**Figure 1:**
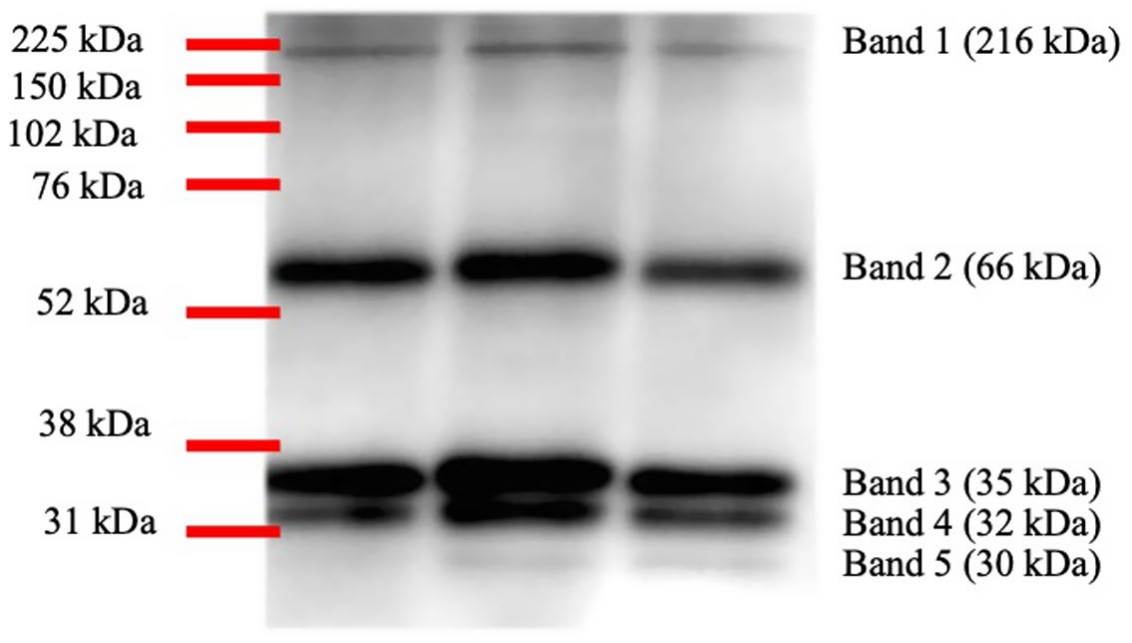
Image representative of the five different bands obtained for salivary carbonic anhydrase VI in porcine saliva samples by Western blotting and the corresponding molecular weights.

A specific signal for CA VI was obtained for five bands in the western blotting analysis corresponding to different molecular weights (Figure 1). The highest intensities were observed at 32 and 35 kDa.

**Figure 2:**
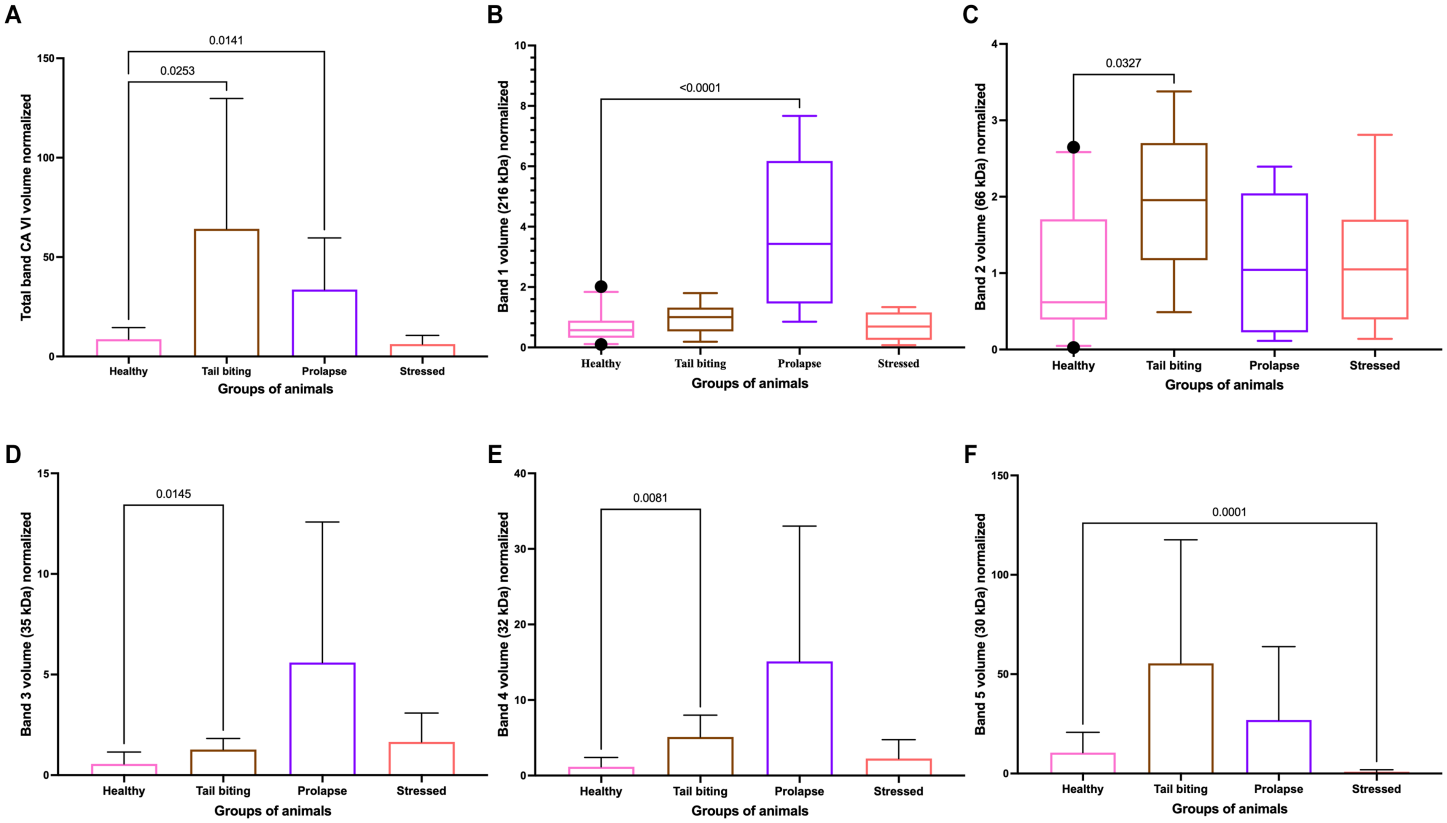
Comparison of the normalised band volume obtained by Western blotting analysis in healthy animals (*n* = 30) and in animals with tail biting (*n* = 10), rectal prolapse (*n* = 10), or acute stress (*n* = 10) for total CA VI **(A)**, band 1 **(B)**, band 2 **(C)**, band 3 **(D)**, band 4 **(E)**, and band 5 **(F)**. The plot shows the mean value and SD (error bars) for the total band and bands 3, 4, and 5. The plot shows the median (line inside the box), 25th and 75th percentiles (box), and 5th and 95th percentiles (whiskers) for bands 2 and 3. The level of significance of the *p*-value is indicated.

The total CA VI signal intensity, quantified as the normalised band volume signal intensity, was statistically significantly higher in the groups of pigs with tail-biting and rectal prolapse in comparison to healthy pigs (Figure 2A). However, the overall signal in the group of stressed pigs did not significantly differ. Similar results were observed when the CA VI signal was analysed in detail in each of the five bands, with no variations in the group of stressed pigs at any band except for the band at 30 kDa, where a statistically significant decrease was observed in stressed animals in comparison to healthy pigs. For the other two pathologies studied, statistically higher values were observed in one or both of the other groups of pigs with pathologies in comparison to healthy animals in at least one of the bands for rectal prolapse and in three bands for tail-biting (Figures 2B–F). The images obtained in the western blottings that were used for band intensity quantification and analyses could be seen in Supplementary Figure S1.

### Field trial

**Figure 3:**
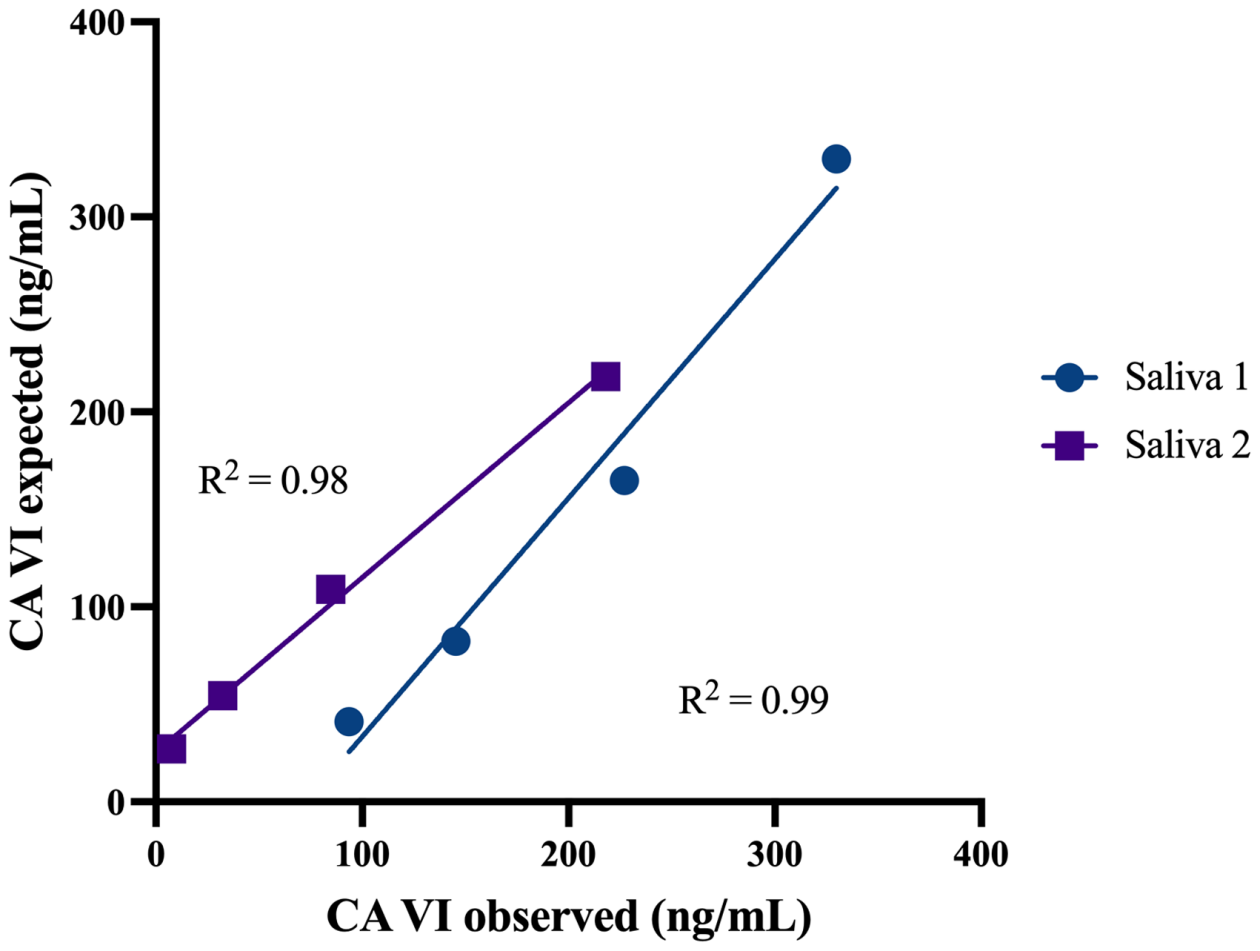
Linear regression lines indicate the accuracy of the assay used to measure salivary CA VI concentrations at serial dilutions of two saliva samples with high salivary CA VI concentrations. The slopes of the regression lines are plotted on the curve as R_2_.

The assay developed for salivary CA VI quantifications showed good precision, with an overall CV of approximately 7% (Table 1), and accuracy, with a coefficient of determination of *R*^2^ = 0.98 (Figure 3). The limit of detection of the assay was established at 4.60 ng/mL of CA VI.

**Table 1:** Intra and inter-assay precisions of the assay developed for CA VI measurements in porcine saliva pooled samples (four samples per pool).

Precision	Mean (ng/mL)	SD	CV
Intra-assay			
High pool 1	679.68	71.91	10.58
High pool 2	613.62	24.39	3.98
Low pool 1	96.67	6.95	7.19
Low pool 2	90.12	6.70	7.44
Overall	370.02	27.49	7.30
Inter-assay			
High pool 1	812.10	94.98	11.70
High pool 2	660.08	41.90	6.35
Low pool 1	98.95	2.84	2.87
Low pool 2	100.23	6.24	6.22
Overall	417.84	36.49	6.78

SD, standard deviation; CV, coefficient of variation.

**Figure 4:**
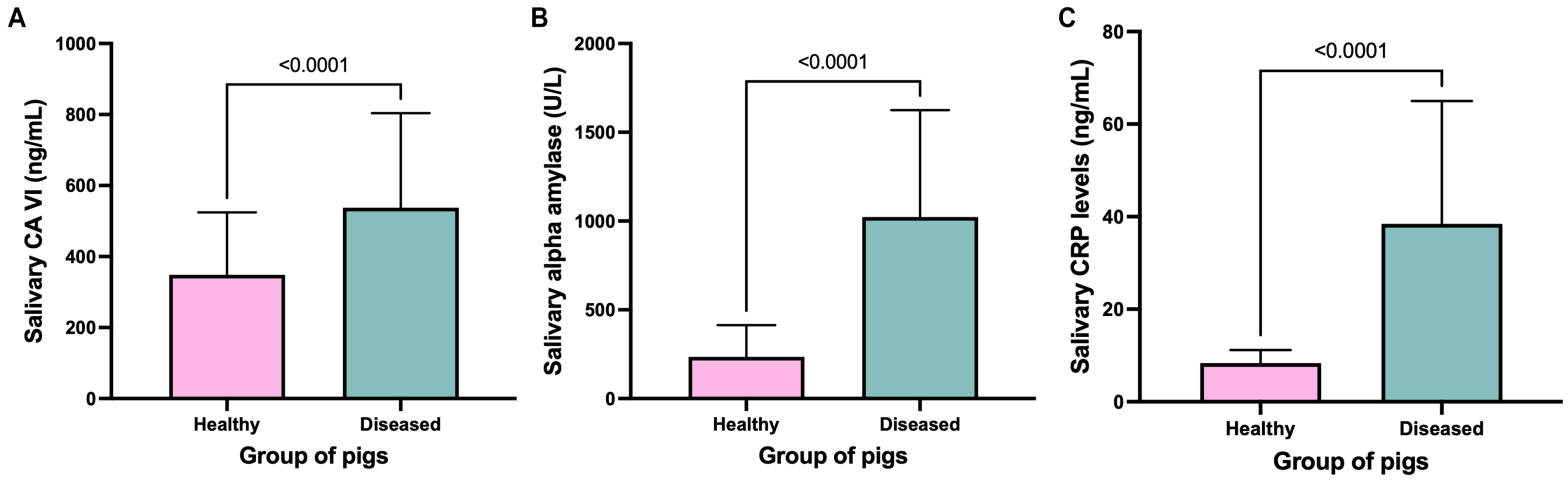
Salivary concentration of CA VI **(A)**, alpha amylase activity level **(B)**, and concentration of CRP **(C)** in healthy and diseased pigs. The plot shows the mean value and SD (error bars). The level of significance of the *p*-value is indicated.

No differences between the levels of CA VI in healthy animals at growing and finishing stages were observed (*p* = 0.18). The overall mean concentration of salivary CA VI in healthy animals was 348.8 ng/mL (SD = 175.8), whilst in diseased pigs the levels increased to a mean value of 551.2 ng/mL (SD = 292.9) with statistical significance (Figure 4A). Similarly, the levels of alpha amylase (mean values of 1,060 U/L ± 661.8 vs. 235.1 U/L ± 178.8, respectively) (Figure 4B) and CRP (mean values of 8.33 ng/mL ± 2.85 vs. 38.44 ng/mL ± 26.57, respectively) (Figure 4C) were statistically higher in diseased animals in comparison to healthy pigs. For all the described comparisons, statistical significance was very high (*p* ≤ 0.0001).

**Figure 5:**
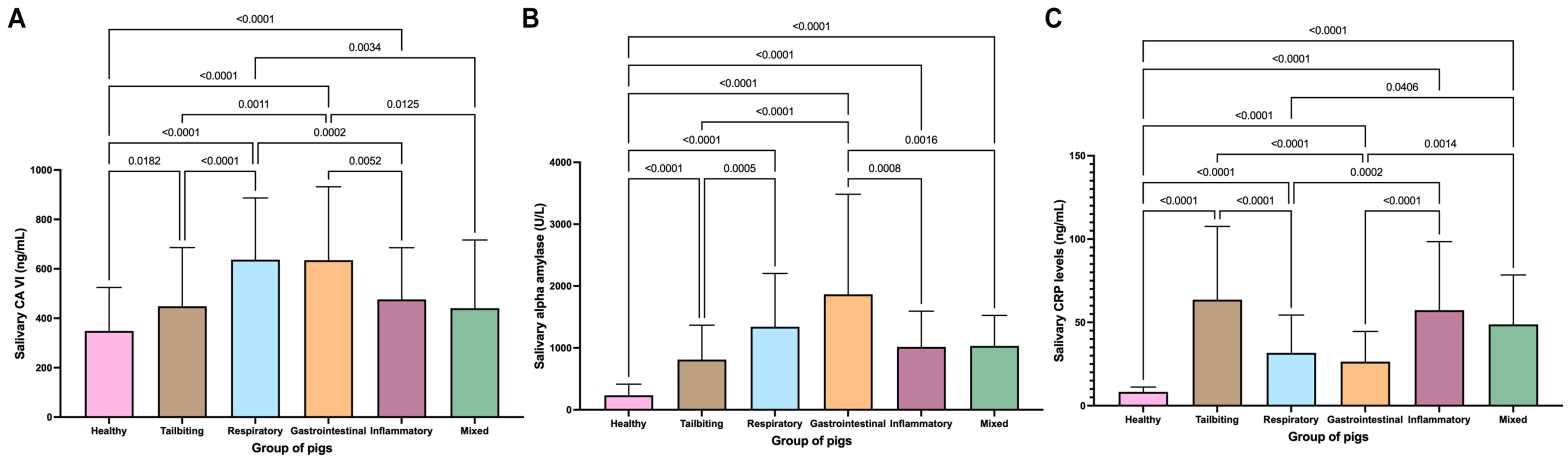
Salivary concentration of CA VI **(A)**, alpha amylase activity level **(B)**, and concentration of CRP **(C)** in healthy pigs and in pigs suffering from tail biting, gastrointestinal disorders, respiratory diseases, inflammatory conditions, or mixed pathologies. The plot shows the mean value and SD (error bars). The level of significance of the *p*-value is indicated.

When diseased pigs were categorised by the type of disease (for descriptive statistics, see Table 2), some differences in the behaviour of the concentrations of salivary parameters were observed. Specifically, for the measurements of salivary CA VI, the highest mean values were reported for animals with gastrointestinal and respiratory diseases, followed by pigs suffering from inflammatory conditions and tail biting, all showing statistically significantly higher values than healthy pigs, whilst the group of pigs with mixed pathologies showed no statistically significant differences with the group of healthy pigs (Figure 5A).

**Table 2:** Descriptive statistics from the quantifications of salivary CA VI, alpha amylase, and CRP in healthy pigs and pigs with different disorders.

Group	*N*	X	SD	Min	Max
CA VI (ng/mL)					
Health	245	348.78	175.77	26.68	829.4
Tail biting	73	448.50	237.25	16.49	857.3
Respiratory	88	637.24	249.49	18.62	1002.1
Gastrointestinal	68	634.84	297.26	16.18	1569.8
Inflammatory	79	476.15	209.32	31.40	941.8
Mixed	41	440.60	276.16	1.43	931.3
Alpha amylase (U/L)					
Health	230	235.10	178.80	22.27	720.5
Tail biting	72	810.61	556.99	90.39	2895.1
Respiratory	69	1340.62	861.55	87.77	3630.0
Gastrointestinal	73	1865.86	1618.04	131.00	6088.9
Inflammatory	78	1016.34	578.24	108.08	2596.4
Mixed	37	1032.27	492.83	155.24	1684.0
CRP (ng/mL)					
Health	247	8.33	2.85	3.96	17.2
Tail biting	66	63.69	43.92	6.78	170.6
Respiratory	80	31.74	22.63	4.56	96.4
Gastrointestinal	73	26.42	18.12	4.30	74.9
Inflammatory	70	57.31	41.16	6.08	167.8
Mixed	38	48.71	29.68	9.69	119.2

*N*, number of animals analysed; X, mean; SD, standard deviation; Min, minimum; Max, maximum.

All the groups of diseased pigs showed statistically significantly higher values of salivary alpha amylase activity in comparison to healthy pigs (Figure 5B). The highest mean levels were observed in the group of pigs with gastrointestinal diseases, followed by pigs with respiratory diseases and pigs suffering from inflammatory conditions or mixed pathologies, whilst the lowest levels were reported for pigs with tail biting.

The concentrations of CRP in all groups of diseases were statistically higher than those of healthy pigs (Figure 5C). The group of pigs with tail biting and inflammatory conditions showed the highest CRP concentrations, followed by the groups of pigs with mixed pathologies; the lowest levels in diseased pigs were observed in the groups of animals with respiratory and gastrointestinal diseases (mean values of 31.74 and 26.42 ng/mL, respectively).

The size effect (represented by the Cohen d value) of the differences between the five diseased groups and healthy pigs for the three biomarkers quantified could be observed in Table 3. The highest Cohen d values were reported in pigs with respiratory diseases for CA VI near the group of pigs with gastrointestinal diseases. Similarly, for alpha amylase levels, the highest size effects were observed in pigs with gastrointestinal disorders, followed by pigs with respiratory diseases, whilst the group with highest size effect for CRP concentrations was the group of pigs with tail biting, followed by pigs with inflammatory conditions.

**Table 3:** Effect size (Cohen’s d) of salivary CA VI, alpha amylase, and CRP in the different groups of diseased animals in comparison to healthy pigs.

Group of diseased pigs	CA VI	Alpha amylase	CRP
Tail biting	0.44	0.78	2.26
Respiratory	1.29	1.49	0.96
Gastrointestinal	1.27	2.20	0.74
Inflammatory	0.57	1.06	2.00
Mixed	0.41	1.08	1.65

A moderate statistically significant positive association between salivary CA VI concentrations and alpha amylase activity levels was detected with a Spearman’s coefficient of variation of r = 0.44, whilst a low statistically significant positive association was observed with CRP (r = 0.24).

## Discussion

In the pathogenesis of porcine diseases, several physiological pathways could be involved, such as acute phase reaction, oxidative stress, inflammation, immune reaction, and even stress, which could be evaluated by the quantification of different general biomarkers in biological fluids such as serum or saliva (25). Nevertheless, saliva is more than a reflection of serum biomarkers (25), as it contains proteins that are involved in the local homeostasis of the mouth and could reflect oral or systemic disturbances (30). Hence, it has been highly recommended to explore saliva as a source of biomarkers of disease status (31).

Carbonic anhydrase VI is a saliva-secreted protein that has been pointed out in several proteomic studies as a potential biomarker candidate under different pathological conditions in the pig. Specifically, in kidney inflammatory diseases (1), acute stress (11), rectal prolapse (12), or growth rate retardation (13). However, authors do not agree about the behaviour of the levels of CA VI under disease conditions, reporting increases or decreases in specific proteoforms. This inconsistency could be based on the microheterogeneity of this protein in saliva (6) and on the absence of proper quantifications of the concentrations of salivary CA VI. Thus, the present study is focused on the study of the overall behaviour of salivary CA VI in stress and disease conditions and on the quantification of the concentrations of CA VI in the saliva of pigs with different health statuses under field conditions.

First, in a small validation trial, we have identified an overall increase in the intensity of salivary CA VI in animals with tail biting and rectal prolapse in comparison to healthy pigs, like the results reported for pigs with growth-rate retardation (13). An intracellular stress-induced form of CA VI has been previously identified in rats, suggesting that the secreted CA VI could be lacking in animals under stress conditions (32). The absence of overall intensity modification observed in our animals subjected to acute stress, with mean values even lower than those of the healthy pigs, might be related to the intracellular form and agree with no alterations in the salivary CA VI levels in chronic-stressed piglets (23).

In a field trial involving a large number of animals, we again observed an overall increase in the concentration of salivary CA VI in diseased animals suffering from different pathological conditions.

The CA VI increase with disease has been previously reported in pigs with kidney disease by the measurement of urinary CA VI concentrations (17). A previous report also indicated a possible increase in salivary CA VI in diseased pigs, specifically in pigs with lameness, based on its involvement in esterase activity (24).

The highest increases in the concentrations of salivary CA VI observed in the present study in pigs with respiratory and gastrointestinal diseases could be related to a protective role against injury in the epithelial cells of these tracts. This hypothesis is supported by previous research in which altered functions have been observed in CA VI-deficient mice, such as immune and catabolic responses in the duodenum and stomach (21) or antigen transport by M-cells in the trachea (22). Moreover, it has been defined that the CA VI found in gastric mucus represents the swallowed protein from the saliva (20) that protects the gastroesophageal mucosa from acid injury (33), similarly to the protective role suggested for the CA VI in the epithelia of the respiratory tract related to susceptibility reduction to bacterial infection (34). The highest increases in the concentrations of CA VI appeared in the same groups of diseased pigs as the highest increases observed in alpha amylase levels, specifically in pigs with gastrointestinal and respiratory disorders. In addition, we have observed an association between the salivary levels of CA VI and alpha amylase, which also agrees with previous reports on human saliva (35), with a similar magnitude of association (0.46 vs. 0.43). These results could indicate that the porcine salivary CA VI is controlled by the autonomic nervous system (36), such as the regulation of salivary alpha amylase levels (37), and future studies should effectively analyse the connection between both proteins in porcine disease.

We have also found an increase in CA VI concentrations in pigs with local inflammatory conditions and tail biting, which could suggest a connection with the innate immune response. The association between CA VI and host defence against infection has been previously defined in mice by a promotion of IL-12 expression in macrophages (38). However, an intracellular form of CA VI and not the secretory CA VI is pointed to as the cause for the immune stimulation. Nevertheless, the study is exclusively focused on the protein expression in macrophages and reports no information about the possible alterations in the levels of salivary CA VI. On the other hand, what we found in the present study, that those diseased groups of pigs, suffering from local inflammatory conditions and tail biting, showed the highest increase in the concentrations of CRP, associated with an acute intense inflammatory condition (26) accompanied with high salivary CA VI levels, could suggest a role of the secretory CA VI in the innate immune response. Further studies would be needed to clarify the connection between salivary CA VI and the inflammatory response.

Our overall data suggest that salivary CA VI has important protective roles in different tracts but also other roles that should be explored in depth, i.e., particularly, but not exclusively, related to the inflammatory response in pigs.

The present study proposed an effective immunoassay for the quantification of the levels of salivary CA VI in pigs, and we expected that additional studies would be performed to understand the specific roles of salivary CA VI in pigs.

## Conclusion

Salivary carbonic anhydrase VI is a biomarker of disease conditions in pigs that could be used for disease detection and characterisation in field conditions if evaluated with additional biomarkers. The concentrations of CA VI are increased under pathological conditions, especially in gastrointestinal and respiratory diseases but also, to a lesser extent, in inflammatory conditions.

## Data Availability

The raw data supporting the conclusions of this article will be made available by the authors, without undue reservation.
