# Supplementary material for: Porcine salivary carbonic anhydrase VI is involved in the pathogenesis of disease
Source: Front Vet Sci. 2026 Jan 7;12:1675415. doi: 10.3389/fvets.2025.1675415 (PMC12819230; doi:10.3389/fvets.2025.1675415)
Supplement: Supplementary file 2 [file Table_2.docx]

Supplementary Material from the manuscript titled: Porcine salivary carbonic anhydrase VI is involved in the pathogenesis of disease.

M. Matas-Quintanilla, R.I. López-Balibrea, I. Miller, A.M. Gutiérrez.

# Supplementary Table S2

**Table S2.** Composition of the diet in percentages (%) in post-weaning pigs and in growing and finishing pigs.

| **Diet** | **Post-weaning** | **Diet** | **Growing** | **Finishing** |
| --- | --- | --- | --- | --- |
| Components |  | Components |  |  |
| Corn | 27.12 | Corn | 34.22 | 33.86 |
| Blonde oatmeal | 1.73 | Sorghum | 4.52 | 3.95 |
| Barley | 15.89 | Barley | 12.85 | 13.62 |
| Soy flour | 9.38 | Soy flour | 9.84 | 10.72 |
| Animal protein | 1.17 | Rye | 1.98 | 2.96 |
| Wheat | 23.93 | Wheat | 20.93 | 21.96 |
| Cookie flour | 4.61 | Sunflower | 1.51 | 1.94 |
| Soy concentrate | 2.26 | Rapeseed flour | 2.26 | 0.51 |
| Lignocellulose | 0.86 | Peas | 2.89 | 0.69 |
| Fish flour | 0.30 | Triticale | 0.00 | 0.59 |
| Turkey flour | 0.29 | Wheat bran | 0.64 | 0.48 |
| Beet pulp | 0.65 | sunflower pellets | 0.44 | 1.20 |
| Enzymes | 0.06 | Palm kernel | 0.82 | 0.68 |
| Corn-wheat-barley flakes | 0.80 | Turkey flour | 0.28 | 0.69 |
| Full fat | 2.02 | Beet pulp | 0.42 | 0.20 |
| Arginine | 0.10 | Palm oil | 0.00 | 0.18 |
| Liquid lysine | 0.57 | Liquid lysine | 0.86 | 0.92 |
| Lard | 2.47 | Lard | 1.47 | 1.44 |
| Dried lysine | 0.32 | Molasses | 0.44 | 0.41 |
| Carbonate | 0.39 | Carbonate | 0.61 | 0.58 |
| Sea salt | 0.36 | Sea salt | 0.39 | 0.37 |
| Vitamin mineral corrector | 0.29 | Vitamin mineral corrector | 0.20 | 0.20 |
| Threonine | 0.28 | Threonine | 0.21 | 0.23 |
| Monocalcium phosphate | 0.72 | Monocalcium phosphate | 0.25 | 0.20 |
| Liquid methionine | 0.32 | Liquid methionine | 0.18 | 0.20 |
| Organic acids | 0.20 | Sodium bicarbonate | 0.15 | 0.15 |
| Water | 1.16 | Water | 0.07 | 0.06 |
| Phytases | 0.05 | Phytases | 0.04 | 0.04 |
| Tryptophan | 0.12 | Tryptophan | 0.05 | 0.05 |
| Valine | 0.21 | Valine | 0.06 | 0.07 |
| Benzoic acid | 0.10 |  |  |  |
| Citric acid anhydride | 0.10 |  |  |  |
| Butyrate | 0.19 |  |  |  |
| Zinc chelates | 0.05 |  |  |  |
| Nutritional value |  | Nutritional value |  |  |
| Water | 11.01 | Water | 11.72 | 11.64 |
| Crude protein | 15.10 | Crude protein | 15.01 | 14.64 |
| Fat | 4.17 | Fat | 3.46 | 3.27 |
| Crude fiber | 5.42 | Crude fiber | 4.35 | 4.47 |
| Ash | 4.48 | Ash | 4.10 | 3.99 |
| Starch | 44.69 | Starch | 47.37 | 47.46 |
| Calcium | 0.79 | Calcium | 0.66 | 0.66 |
| Phosphorus | 0.34 | Phosphorus | 0.36 | 0.36 |
