# Supplementary material for: Porcine salivary carbonic anhydrase VI is involved in the pathogenesis of disease
Source: Front Vet Sci. 2026 Jan 7;12:1675415. doi: 10.3389/fvets.2025.1675415 (PMC12819230; doi:10.3389/fvets.2025.1675415)
Supplement: Supplementary file 3 [file Supplementary_file_1.docx]

Supplementary Material from the manuscript titled: Porcine salivary carbonic anhydrase VI is involved in the pathogenesis of disease.

M. Matas-Quintanilla, R.I. López-Balibrea, I. Miller, A.M. Gutiérrez.

# Supplementary Figure S1


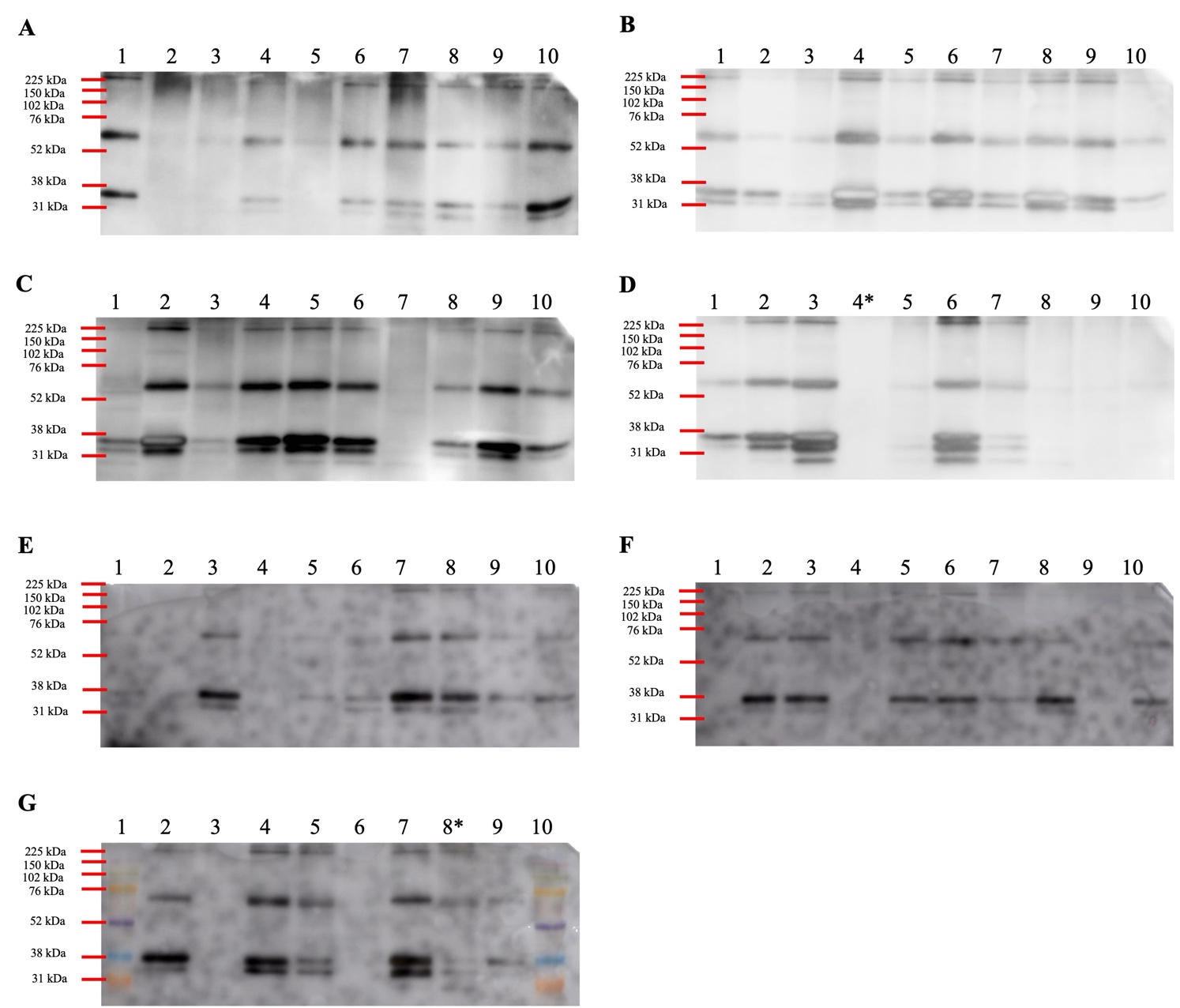


**Figure S1**. CA VI bands in the saliva of healthy pigs (lanes 1-9 from A, C & E and lanes 2, 4 & 6 from G), pigs with tail biting (lane 1-9 from B and lane 3 from G), rectal prolapse (lanes 1-9 from D and lane 5 from G) and pigs under acute stress (lanes 1-9 from f and lane 7 from G) and in a control sample (lane 10 from A-F and lane 9 from G). The asterisk highlights the repetition of the lane 4 from D in lane 8 from G. Molecular weight markers (kDa): Lane 1 & 10 from G.
